# 366. Optimization of a Surrogate Assay for Efficacy of a Malaria Transmission Blocking Vaccine

**DOI:** 10.1093/ofid/ofac492.444

**Published:** 2022-12-15

**Authors:** Cristina Meehan, Cristina Meehan, Patrick Duffy, Jonathan Renn, Irfan Zaidi

**Affiliations:** National Institutes of Health, National Institute of Allergy and Infectious Diseases, Bethesda, Maryland; National Institutes of Health, National Institute of Allergy and Infectious Diseases, Bethesda, Maryland; National Institutes of Health, National Institute of Allergy and Infectious Diseases, Bethesda, Maryland; National Institutes of Health, National Institute of Allergy and Infectious Diseases, Bethesda, Maryland; National Institutes of Health, National Institute of Allergy and Infectious Diseases, Bethesda, Maryland

## Abstract

**Background:**

An estimated 241 million malaria cases and 627,000 malaria deaths occurred worldwide in 2020. Efforts to develop a higher efficacy malaria vaccine are a global priority, but parasites pose a complex biological puzzle for vaccine development. Innovative vaccine strategies include transmission blocking vaccines (TBV) that target the mosquito stages of parasite development to interrupt parasite transmission. The current leading TBV called Pfs230D1-EPA targets Pfs230, a Plasmodium falciparum gamete surface protein that mediates binding of microgametes to red blood cells. Antibodies against Pfs230 bind and lyse gametes in a complement-dependent manner to block transmission.

**Methods:**

Here, we are exploiting a panel of human monoclonal antibodies (hmAb) generated from human volunteers who received Pfs230D1-EPA formulated in Alhydrogel or AS01 adjuvants, to develop a competitive ELISA platform. This assay will quantify epitope-specific serum antibodies along with their features and effector functions, expanding the conventionally used functional assay of standard membrane feeding (SMFA).

**Results:**

The competitive ELISA is being developed with single-chain variable fragments (ScFv) to define epitope-specific binding of antibodies in sera from vaccinated individuals. Sera of human volunteers from trials in Mali, Africa will be assayed to assess epitope-specific serum antibody binding that is competed off in the presence of saturating levels of ScFv. Preliminary optimization of the competitive assay has shown concentration-dependent epitope-specific competition between ScFv and hmAbs and confirmation of ScFv binding to Pfs230D1 antigen.

**Conclusion:**

Further optimization of this assay will characterize the complement-binding and isotype/subclass-specific features of epitope-specific antibodies. These efforts will lay the foundations for standardized assays that can assess correlates of vaccine efficacy and durability of this leading TBV.

**Disclosures:**

**All Authors**: No reported disclosures.

